# Oligonucleotide conjugated multi-functional adeno-associated viruses

**DOI:** 10.1038/s41598-018-21742-x

**Published:** 2018-02-26

**Authors:** Dhruva Katrekar, Ana M. Moreno, Genghao Chen, Atharv Worlikar, Prashant Mali

**Affiliations:** Department of Bioengineering, University of California, San Diego, CA USA

## Abstract

Recombinant adeno-associated viruses (AAVs) are among the most commonly used vehicles for *in vivo* gene delivery. However, their tropism is limited, and additionally their efficacy can be negatively affected by prevalence of neutralizing antibodies in sera. Methodologies to systematically engineer AAV capsid properties would thus be of great relevance. In this regard, we develop here multi-functional AAVs by engineering precision tethering of oligonucleotides onto the AAV surface, and thereby enabling a spectrum of nucleic-acid programmable functionalities. Towards this, we engineered genetically encoded incorporation of unnatural amino acids (UAA) bearing bio-orthogonal chemical handles onto capsid proteins. Via these we enabled site-specific coupling of oligonucleotides onto the AAV capsid surface using facile click chemistry. The resulting oligo-AAVs could be sequence specifically labeled, and also patterned in 2D using DNA array substrates. Additionally, we utilized these oligo conjugations to engineer viral shielding by lipid-based cloaks that efficaciously protected the AAV particles from neutralizing serum. We confirmed these ‘cloaked AAVs’ retained full functionality via their ability to transduce a range of cell types, and also enable robust delivery of CRISPR-Cas9 effectors. Taken together, we anticipate this programmable oligo-AAV system will have broad utility in synthetic biology and AAV engineering applications.

## Introduction

Adeno associated viruses (AAVs) are ~4.7 kb single-stranded DNA viruses that infect humans and primates^1^. Belonging to the *Parvoviridae* family, the AAV classifies as a *Dependoparvovirus* genus, due to its dependence on helper viruses such as adenoviruses to complete its replicative cycle^2^. AAVs are among the most commonly used vectors for gene delivery, with a number of clinical trials showing promising results, including for Duchenne Muscular Dystrophy^3^, Lipoprotein Lipase (LPL) deficiency^4,5^ and Hemophilia B^6–8^ among others. Their lack of pathogenicity and ability to infect both dividing and non-dividing cells, while providing persistent levels of transgene expression makes them favorable systems for *in vivo* gene transfer. However, several challenges still need to be overcome to enable their widespread use as vectors for gene delivery, such as limited packaging capacity^9^, immunogenicity^10–13^, restricted tropism, and tissue specificity^14^. Furthermore, efficiency of delivery is limited by the fact that widespread exposure to AAVs has resulted in a significant portion of the human population harboring neutralizing antibodies against many of the natural AAV serotypes^10–13^. Engineering precise surface modifications on the AAVs, towards enabling tissue specific targeting and altering tropism or evasion of the immune system would thus be hugely beneficial for enabling efficient *in vivo* gene transfer. However, engineering such modifications is challenging as insertion of large peptides or biomolecules into AAV capsid proteins often results in significant loss of titer or functionality^15–18^.

**Figure 1:**
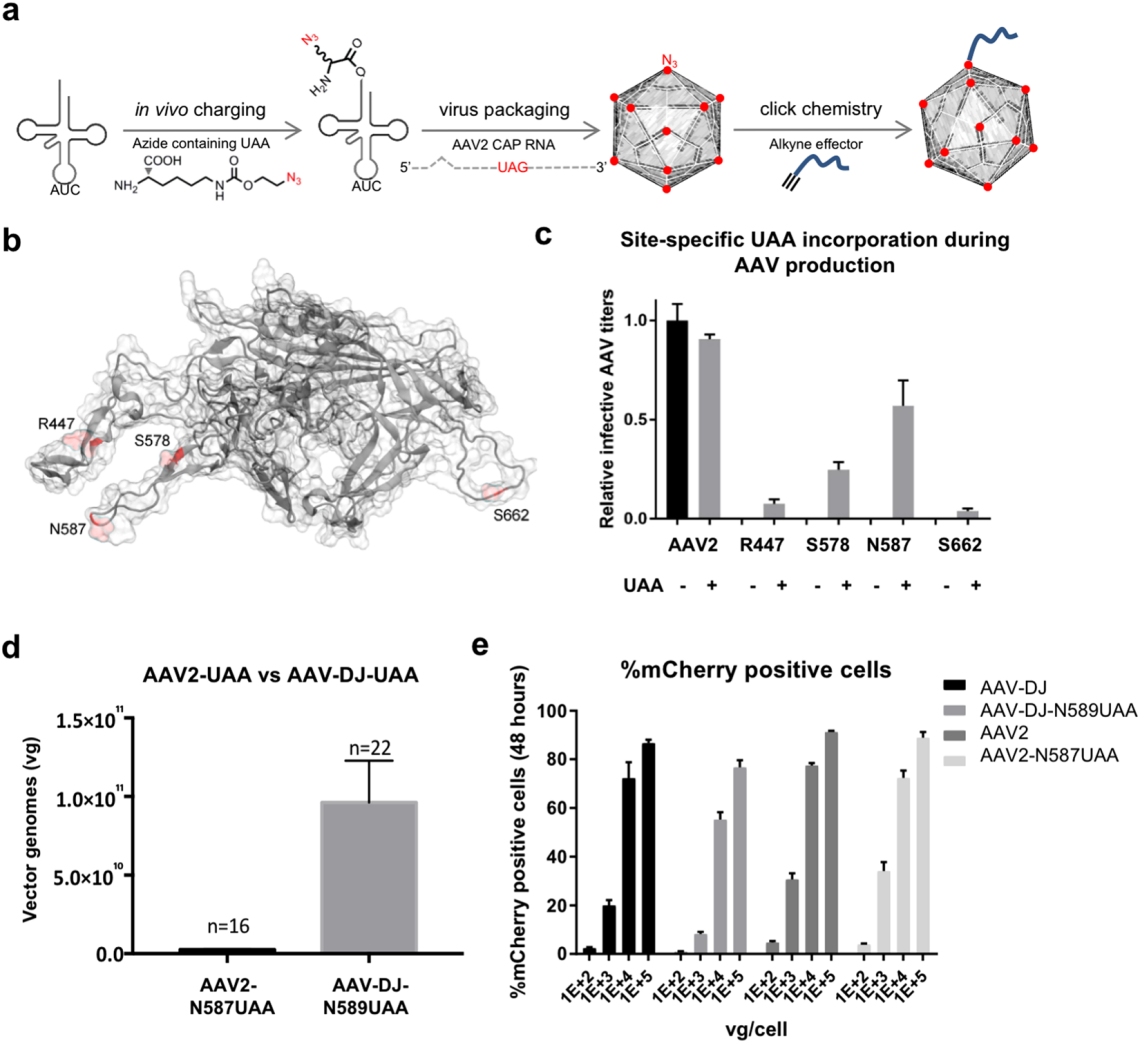
Engineering robust UAA incorporation into AAVs. (**a**) Schematic of approach for addition of an azide bearing UAA to the virus capsid and subsequent click-chemistry based chemical linking of an effector to the UAA. (**b**) Locations of the surface residues assayed for replacement with UAAs (VP1 residues numbered). (**c**) Relative infective titers of the AAV2 mutants in the presence and absence of 2 mM UAA, quantified via the transduction of HEK293T cells and subsequent mCherry exprssion (n = 3 independent replicates, cells transduced with equal volumes of virus) (Error bars are SEM). (**d**) Comparison of the viral titers of AAV2-N587UAA and AAV-DJ-N589UAA (Error bars are SEM). (**e**) Confirmation that UAA incorporation does not negatively affect AAV activity (n = 3 independent replicates; experiments performed in HEK293Ts at varying vector genome containing particles (vg)/cell) (Error bars are SEM).

Towards addressing these challenges, capsid engineering offers great potential and there are at least four major techniques currently used towards this: rational engineering^16–19^, directed evolution^20–22^, evolutionary lineage analysis^23^, and chemical conjugation^24–27^. Rational capsid engineering combines the knowledge from AAV structural analysis with insights from other aspects such as delivery mechanisms, cell surface receptors, or neutralizing antibodies, to engineer AAVs with favorable characteristics. For example, rational capsid engineering has been used to generate tumor targeting AAV variants among others^28^. In comparison, directed evolution involves the generation of capsid libraries by genetic shuffling or mutagenesis followed by extensive screening to isolate variants with desired features. For instance, this approach was applied to the problem of neutralizing antibodies and resulted in generation of AAV2 variants that could withstand significantly higher levels of neutralizing antibodies than the natural serotype^20,21^. Evolutionary lineage analysis involves *in silico* ancestral sequence reconstruction of AAV capsid proteins, followed by de novo synthesis and screening of the ancestral AAVs. This novel method has recently been successfully utilized to predict an ancestor of commonly studied AAV serotypes 1, 2, 8 and 9 that is highly potent in targeting liver, muscle and retina^23^. Albeit excellent approaches, these methods cannot however be used for incorporation of small molecules or aptamers that are not genetically encoded. In this regard, chemical conjugation based approaches could be used to enable this aspect^24,25^. However, chemical conjugation based approaches for the modification of AAVs rely primarily on surface exposed arginine or lysine residues, and lack specificity and can impact viral stability^26^. Here we build on a recent approach to incorporate unnatural amino acids (UAAs) on the AAV capsid that bear bio orthogonal chemical handles^27^. Specifically, we expand this approach to enable surface programmable AAVs across multiple serotypes, selectively focusing on UAA incorporation without loss of AAV activity and generation of functional oligo-AAVs. We genetically encode chemical handles via these UAAs on to the capsid surface of AAV2 and AAV-DJ, which in turn can be used to covalently attach synthetic effector molecules, in particular oligonucleotides to leverage nucleic acid programmability, thereby imparting new features to the AAVs (Fig. 1a). We also combine UAA-mediated capsid engineering and oligonucleotide tethering with robust CRISPR payload delivery, and further utilize the coupled oligonucleotides to engineer lipid based shielding of AAVs from neutralizing antibodies in sera. Taken together, we anticipate this programmable oligo-AAV system will have broad utility in synthetic biology and AAV engineering applications.

## Results

The AAV capsid is a self-assembled structure, made up of three capsid proteins - VP1, VP2 and VP3 in a 1:1:10 molar ratio, for a total of 60 subunits which are arranged in an icosahedral symmetry, with a triangulation number of 1^29^. These capsid proteins are transcribed from the *cap* gene and contain overlapping C-terminal residues. Leveraging the availability of the AAV2 crystal structure, we computationally identified accessible amino acid residues on the surface of the AAV2 as potential candidates for UAA incorporation. Based on this, we focused on the residues R447, S578, N587 and S662 (VP1 numbering scheme) (Fig. 1b)^29^. Positions S578 and N587 are located in the basic cluster that the AAV uses to bind a key cellular receptor, Heparan Sulfate Proteoglycan (HSPG), while residues R447 and S662 are surface residues located in the putative loop regions of VP3^15–18^. Since the use of orthogonal translation systems has made UAA incorporation possible at reassigned stop codons^30,31^, these capsid residues were individually replaced with the amber stop codon, TAG. Next, we co-translationally incorporated an UAA, specifically an azide modified pyrrolysine derivative - N-epsilon-((2-Azidoethoxy)carbonyl)-L-lysine (Supplementary Figure 1a) into the three capsid proteins VP1–3 at the amber stop codon, by utilizing the orthogonal pyrrolysyl tRNA/aminoacyl-tRNA synthetase (tRNA/aaRS) pair^32–34^. Towards this we optimized UAA incorporation using a GFP-Y39TAG reporter (Supplementary Figure 1b, 2a, b). The azide modified pyrrolysine derived UAA was chosen for incorporation to leverage the fact that it can undergo an azide-alkyne Huisgen cycloaddition (click chemistry) reaction with an alkyne conjugated effector^35,36^. Three days post transfection, the relative infective titers of the mutants carrying the mCherry transgene, both in the presence and absence of 2 mM UAA, were measured by the transduction of HEK293T cells and quantifying the mCherry positive cells. No infective AAV2 mutants were produced in the absence of the UAA (Fig. 1c). Since the UAA incorporation machinery competes with the endogenous machinery for translation termination, we also co-transfected an Eukaryotic Translation Termination Factor 1 (eTF1) mutant (E55D) which resulted in a further 3 fold increase in AAV titers (Supplementary Figure 2c)^33^.

To facilitate a broader usage of the system, especially for *in vivo* applications requiring high viral titers, we next focused on engineering the AAV-DJ capsid. Created using the method of molecular evolution via DNA family shuffling, the AAV-DJ is a chimera of AAV serotypes 2, 8 and 9, with 92% sequence homology to AAV2^37,38^. Compared to AAV2, AAV-DJ yields ~10 fold higher titers and it outperforms AAV2 in the transduction of multiple cell lines as well as in the livers of naïve mice^38^. Since we found the infectivity of the AAV2-N587UAA mutant to be similar to the wild-type AAV2, we created a corresponding AAV-DJ-N589UAA version. As anticipated, the AAV-DJ-N589UAA exhibited 5 to 15-fold higher titers than the AAV2-N587UAA (Fig. 1d). As with the AAV2-N587UAA, the incorporation of an UAA at the AAV-DJ-N589 residue similarly did not significantly alter AAV infectivity (Fig. 1e).

**Figure 2:**
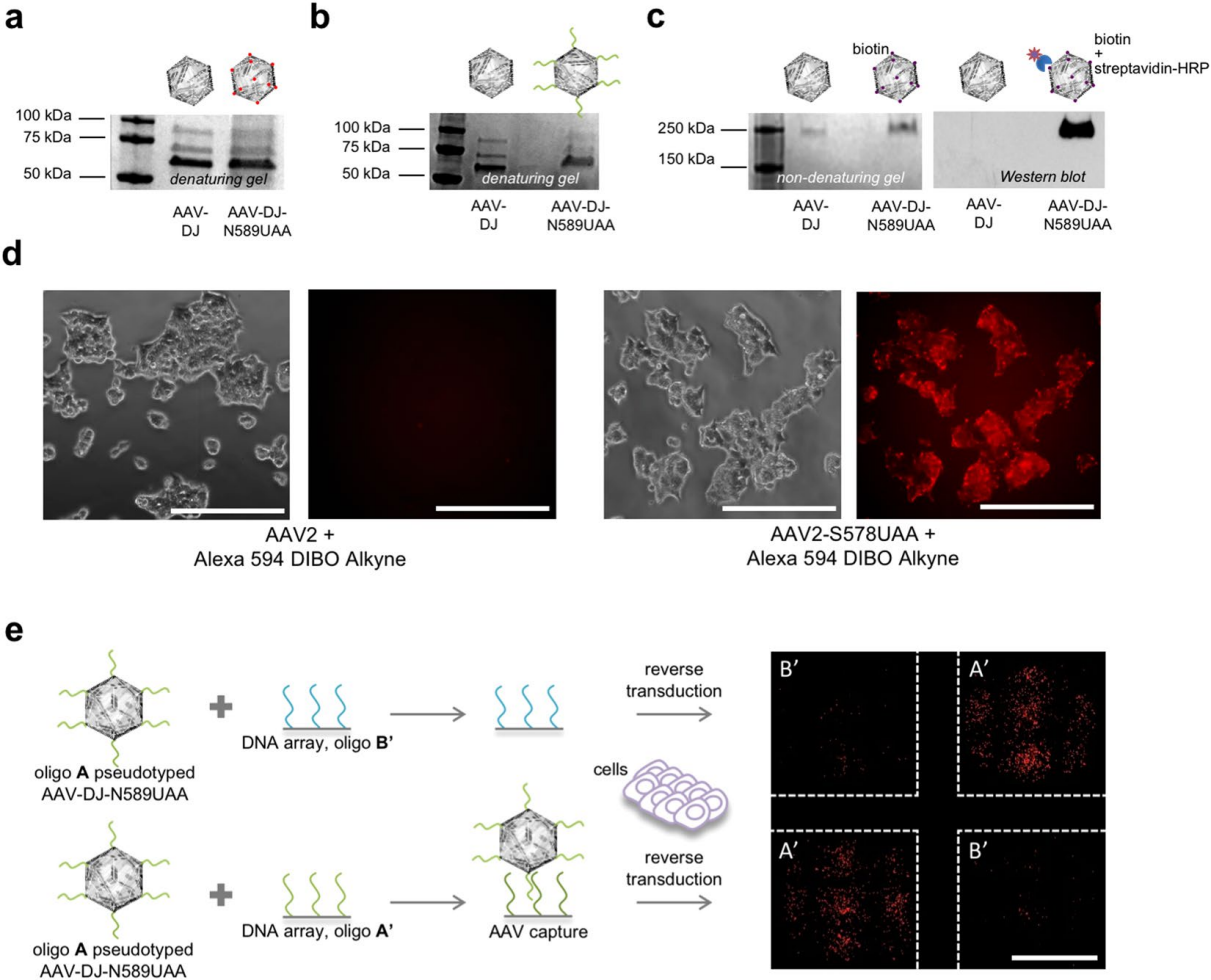
Confirmation of UAA-AAV functionality, and site-specific oligonucleotide conjugation onto UAA-AAVs. (**a**) Coomassie stain of SDS-PAGE resolved capsid proteins of AAV-DJ and AAV-DJ-N589UAA. (**b**) Coomassie stain of SDS-PAGE resolved capsid proteins of AAV-DJ and AAV-DJ-N589UAA following treatment with an alkyne-oligonucleotide (10 kDa). (**c**) Coomassie stain and western blot of the non-denatured AAV-DJ and AAV-DJ-N589UAA following treatment with an alkyne-oligonucleotide, and probed with a complementary oligonucleotide-biotin conjugate followed by streptavidin-HRP. (**d**) Fluorophore pseudotyping of AAVs via Alexa594 DIBO alkyne: successful linking onto the virus confirmed via fluorescence visualization of the virus 2 hours post addition of AAVs onto HEK293T cells (scale bars: 250 μm). (**e**) Oligonucleotide pseudotyping of AAVs via alkyne-tagged oligonucleotide: selective capture on DNA spots (arrayed in a checkerboard fashion) of AAVs bearing corresponding complementary oligonucleotides, evidenced via specific viral transduction of HEK293T cells dispersed on those spots (scale bars: 250 μm).

Next, to confirm UAA incorporation and engineer new functionality on the AAV-DJ capsid, both the wild-type AAV-DJ and the AAV-DJ-N589UAA were treated with an alkyne bearing oligonucleotide (10 kDa) in the presence of copper (Cu^+2^) and then resolved via SDS-PAGE, followed by Coomassie staining of the gel (Fig. 2a, b). We observed a shift in band size of all the three capsid proteins of AAV-DJ-N589UAA, but not wild-type AAV-DJ, confirming the presence of an azide moiety in all the three capsid proteins as well as the successful tethering of the oligonucleotide onto the capsid proteins. In addition, we also resolved the alkyne-oligonucleotide treated wild-type AAV-DJ and AAV-DJ-N589UAA on a non-denaturing gel, transferred it to a nitrocellulose membrane and utilized a complementary oligonucleotide conjugated to biotin as a primary probe. Upon the addition of streptavidin-HRP, a band was obtained only in the case of the UAA modified AAV but not the wild-type AAV-DJ (Fig. 2c). Both these assays not only confirmed the incorporation of the UAA, N-epsilon-((2-Azidoethoxy)carbonyl)-L-lysine, onto the AAV capsid but also confirmed successful oligonucleotide tethering. Next, we treated the wild-type AAV2 and the AAV2-S578UAA with a strained alkyne-fluorophore, Alexa 594 DIBO alkyne, and added the conjugated viruses to HEK293T cells. 2 hours post addition of the AAVs, fluorescence imaging confirmed accumulation of fluorescent particles on the HEK293T cells only for AAV2-S578UAA (Fig. 2d). This confirmed the azide moiety was exposed on the surface of the AAV particles and accessible for chemical modification. Furthermore, leveraging the ability of the DNA in the oligo-AAV to undergo sequence-specific hybridization, we tethered an alkyne-oligonucleotide (label-A) (Supplementary Table 1a) onto the AAV-DJ-N589UAA delivering the mCherry transgene and added the oligonucleotide A pseudotyped AAV to a DNA array bearing complementary and non-complementary oligonucleotides label-A’ and label-B’ respectively, in a checkerboard arrangement. Indeed, we observed selective capture of the oligonucleotide A pseudotyped AAVs only on DNA array spots bearing complementary oligonucleotide A’, as visualized by the mCherry expression in HEK293T cells cultured on corresponding array spots (Fig. 2e). Together, these experiments confirmed the precise incorporation of UAAs with a functional azide group onto the surface of AAVs (hereafter referred to as UAA-AAVs), and the subsequent successful functional coupling of oligonucleotides via UAA mediated click chemistry to generate oligo-AAVs.

**Figure 3:**
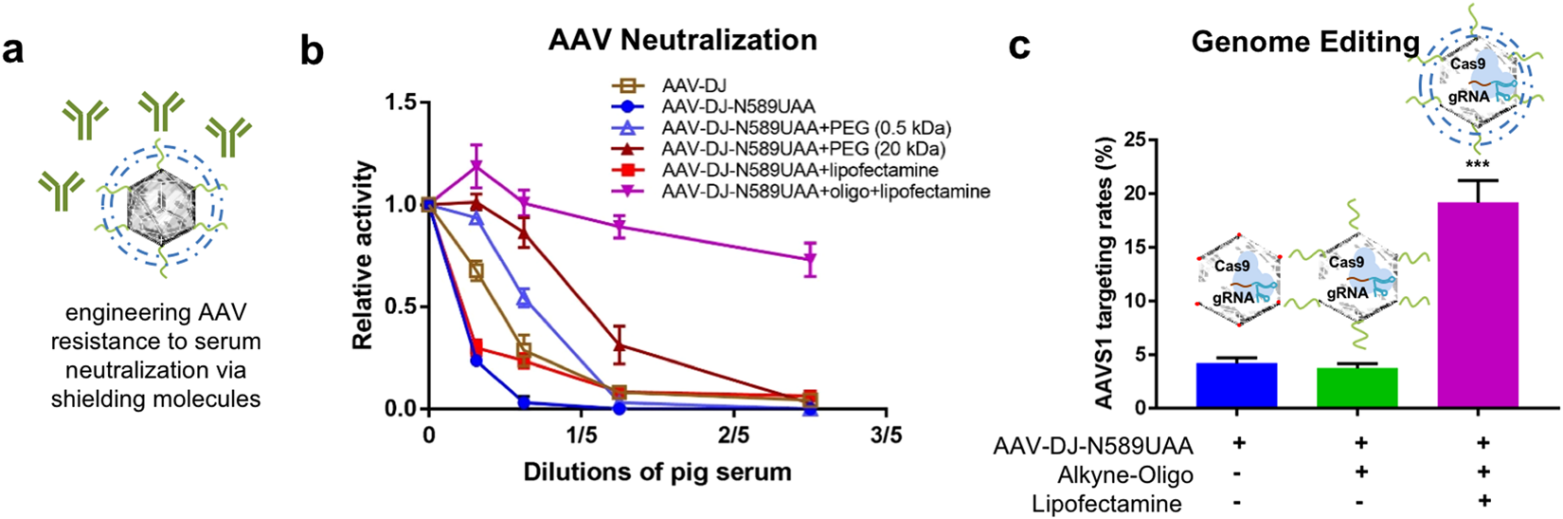
Engineering ‘cloaked AAVs’ resistant to neutralization via sera, and their functional characterization via CRISPR-Cas9 genome editing. (**a**) Representation of a ‘cloaked AAVs’ resistant to antibody neutralization. (**b**) Relative activity of AAVDJ and AAVDJ-N589UAA viruses tethered to a range of small molecule and polymer moieties post exposure to pig serum assayed via AAV-mCherry based transduction of HEK293T cells (n = 4 independent replicates) (error bars are SEM). (**c**) *AAVS1* editing rates (% NHEJ events) of AAV-DJ-N589UAA, AAV-DJ-N589UAA + oligo, and AAV-DJ-N589UAA + oligo + lipofectamine in HEK293T cells (1E + 5 vg/cell) (error bars are SEM).

As prevalence of AAV neutralizing antibodies in sera is a major obstacle to the effective use of AAVs as vectors for gene delivery *in vivo*, we next employed our system to engineer surface modifications that enable resistance to serum based antibodies (Fig. 3a). Towards creating such a ‘cloaked AAV’, we utilized the UAA based chemical handles on the AAV surface to tether a host of alkyne conjugated small molecules and polymers^39,40^ and assayed their effectiveness in cloaking the AAVs from neutralizing antibodies present in pig serum (Supplementary Figure 3a)^41^. Specifically, following click chemistry with a variety of moieties, the pseudotyped AAVs bearing a mCherry transgene, were incubated with pig serum prior to the transduction of HEK293T cells. These were then quantified for mCherry expression after 72 hours. We observed that although 0.5 kDa and 4 kDa polyethylene glycol (PEG) molecules provided moderate shielding against the neutralizing antibodies, they proved to be ineffective at higher concentrations of pig serum. Instead, lipofectamine coated oligo-AAVs demonstrated robust activity across the full range of pig serums. To create these lipid-coupled oligo-AAVs, oligonucleotides were first tethered onto the AAV-DJ-N589UAA via click-chemistry and the oligonucleotide-pseudotyped AAVs were then incubated with lipofectamine, a commercial lipid based transfection reagent. The oligonucleotide pseudotyping was an essential step as it provided the net negative charge that enables lipofectamine complexing. This resulted in the formation of a ‘cloaked AAV’ that retained complete activity at pig serum concentrations, while the wild-type AAV-DJ, AAV-DJ-N589UAA and AAV-DJ-N589UAA + lipofectamine were completely neutralized (Fig. 3b). Incubation of the cloaked AAVs with 0.075% Triton X-100 led to a downward shift in the neutralization curve, indicating some degree of disruption of the lipofectamine shield in presence of the detergent (Supplementary Figure 3b). We also compared the transduction efficiencies of AAV-DJ-N589UAA and the ‘cloaked AAV’ in a variety of cell lines and observed equal or moderately increased transduction efficiency across most tested cell lines (Supplementary Figure 4a) and no overt cytotoxicity in HEK-293-Ts (Supplementary Figure 4b). As AAVs are commonly utilized as gene delivery vehicles, we next also confirmed the ability of the UAA-AAVs, oligo-AAVs and ‘cloaked AAVs’ to deliver CRISPR effectors. Towards this we packaged a split-SpCas9 system^42,43^ into the modified AAVs and tested them *in vitro*, by targeting the endogenous *AAVS1* locus in HEK293-Ts, and indeed achieved robust editing rates (Fig. 3c, Supplementary Figure 5a, b). These experiments confirm that UAA incorporation does not compromise the ability of the modified AAVs to package large DNA payloads. Furthermore, the ‘cloaked AAVs’ displayed nearly 4-fold higher *AAVS1* editing rates than the UAA-AAVs.

## Discussion

Collectively, we believe that our oligo-AAV approach paves the way for programmable control of AAV surface properties, thereby enabling a systematic evaluation of effectors for engineering AAVs with novel surface properties. Specifically, we demonstrated the successful incorporation of an UAA on the surface of the AAV and used it to couple oligonucleotides, and notably further engineered ‘cloaked AAVs’ that are resistant to serum based neutralizing antibodies. We also incorporated a split-Cas9 system into the UAA-AAVs, oligo-AAVs, and ‘cloaked AAVs’ and demonstrated robust editing of the endogenous *AAVS1* locus. Future studies will focus on the lipid based shielding mechanism of the ‘cloaked AAVs’ to better understand the shielding mechanism. Furthermore, altered cellular entry, lipid based cytotoxicity, and its impact on AAV functionality will need to be systematically evaluated. *In vivo* studies to understand the stability and tropism of the ‘cloaked AAVs’ as well as their neutralization kinetics will also be critical in this regard. We recognize a potential limitation of our system, in that, we observe UAA based capsid modifications typically lead to 1.5–5 fold lower viral titers. With optimization of AAV production parameters we however anticipate these aspects will be progressively addressed.

Taken together, we believe that this programmable system opens the door for the site-specific addition of a spectrum of molecules that can be employed for modulation of AAV activity. Tethering of oligonucleotides will enable us to leverage properties of DNA aptamers which have a high affinity for molecules such as proteins, nucleotides, drugs, and other small molecules^44^. These oligonucleotides could potentially be used towards overcoming the problem of the host immune response such as by enabling AAV shielding against neutralizing antibodies as demonstrated here. They could also be used to achieve tissue specific AAV targeting by taking cues from aptamer-nanoparticle or other aptamer-virus conjugates that have been used to selectively target specific cell types^45–47^. As the UAA-AAV technology and oligonucleotide coupling combines the benefits of genetic and non-genetic capsid engineering approaches, and also the potential of leveraging DNA nanotechnology methodologies^48–51^, we believe that this platform will find broad applications both in basic studies of AAV biology as well as in AAV engineering applications.

## Materials and Methods

### Vector design and construction

Four gene blocks were synthesized with ‘TAG’ inserted in place of the nucleotides coding for the AAV2 capsid residues R447, S578, N587 and S662, and the AAV-DJ capsid residue N589 and were inserted into the pAAV-RC2 and pAAV-DJ vectors (Cell Biolabs) respectively using Gibson assembly. For ETF1-E55D, the gene block encoding the protein sequence was synthesized and inserted downstream of a CAG promoter via Gibson assembly.

### Mammalian cell culture

All HEK293T cells were grown in Dulbecco’s Modified Eagle Medium (10%) supplemented with 10% FBS and 1% Antibiotic-Antimycotic (ThermoFisher Scientific) in an incubator at 37 °C and 5% CO_2_ atmosphere. HEK293T cells were plated in in 24-well plates for AAV transductions.

### Production of AAVs

#### Large-scale production of UAA-AAVs

AAV2/DJ virus particles were produced using HEK293T cells via the triple transfection method and purified via an iodixanol gradient^52^. The AAV2/DJ mutants were produced via 5 plasmid transfections. Confluency at transfection was between 80% and 90%. Two hours prior to transfection, DMEM supplemented with 10% FBS and 2mM N-epsilon-((2-Azidoethoxy)carbonyl)-L-lysine was added to the HEK293T cells. Each virus was produced in 5 × 15 cm plates, where each plate was transfected with 7.5 μg of pXR-capsid (pAAV-RC2/DJ mutants), 7.5 of μg recombinant transfer vector, 22.5 μg of pAd5 helper vector, 7.5 μg of pCAG-eTF1-E55D and 22.5 μg of pAcBac1.tR4-MbPyl (gift from Peter Schultz, Addgene #50832) containing the pyrrolysyl-tRNA and tRNA synthetase, using PEI (1 μg/uL linear PEI in 1xDPBS pH 4.5, using HCl) at a PEI:DNA mass ratio of 4:1. The mixture was incubated for 10 minutes at RT and then applied dropwise onto the cells media. The virus was harvested after 72 hours and purified using an iodixanol density gradient ultracentrifugation method. The virus was then dialyzed with 1 × PBS (pH 7.2) supplemented with 50 mM NaCl and 0.0001% of Pluronic F68 (Thermo Fisher) using 50 kDA filters (Millipore), to a final volume of ~1 mL and quantified by qPCR using primers specific to the ITR region, against a standard (ATCC VR-1616).

*AAV-ITR-F: 5′-CGGCCTCAGTGAGCGA-3′ and*

*AAV-ITR-R: 5′-GGAACCCCTAGTGATGGAGTT-3′*.

To further quantify functional activity, flow cytometry analysis of HEK293T cells transduced with UAA-AAVs delivering mCherry, was performed 48 hours post transduction using a FACScan Flow Cytometer and the Cell Quest software (both Becton Dickinson).

#### Small-scale production

Small-scale AAV production was carried out in 6-well plates containing HEK293T cells, which were co-transfected with 0.5 μg pAAV-RC2/DJ mutants, 0.5 μg recombinant transfer vector, and 1.5 μg pAd5 helper vector, 0.5 μg of pCAG-eTF1-E55D and 1.5 μg of pAcBac1.tR4-MbPyl using PEI. The cells and supernatant were harvested after 72 hours, and the crude extract was utilized to transduce HEK293T cells in 24 well plates.

### Genomic DNA extraction and NGS

gDNA from cells was extracted using DNeasy Blood and Tissue Kit (Qiagen), according to the manufacturer’s protocol. Next generation sequencing libraries were prepared as follows. Briefly, 4–10 μg of input gDNA was amplified by PCR with primers that amplify 150 bp surrounding the sites of interest (Supplementary Table 1b) using KAPA Hifi HotStart PCR Mix (Kapa Biosystems). PCR products were gel purified (Qiagen Gel Extraction kit), and further purified (Qiagen PCR Purification Kit) to eliminate byproducts. Library construction was done with NEBNext Multiplex Oligos for Illumina kit (NEB). 10–25 ng of input DNA was amplified with indexing primers. Samples were then purified and quantified using a qPCR library quantification kit (Kapa Biosystems, KK4824). Samples were then pooled and loaded on an Illumina Miseq (150 bp paired-end run or 150 single-end run) at 4 nM concentrations. Data analysis was performed using CRISPR Genome Analyzer^53^.

### AAV pseudotyping

#### Alexa 594 DIBO alkyne tethering

The AAV2/AAV-DJ wild-type and AAV2-S578UAA/AAV-DJ-N589UAA were incubated with Alexa 594 DIBO alkyne^54^ in TBS (both ThermoFisher Scientific) for 1 hour at room temperature. The excess label was washed off with PBS. The virus particles were added to HEK293T cells and the cells were imaged 2 hours post addition of the AAVs.

#### Oligonucleotide tethering and DNA array

Oligos A’ and B’ (5 uM) were spotted on a streptavidin functionalized array (ArrayIt: SMSFM48) and incubated at room temperature for 30 minutes^55^. Meanwhile, oligo A was linked to AAV2-N587UAA_mCherry/AAV-DJ-N589UAA_mCherry via the process of click chemistry (Click-iT – ThermoFisher Scientific, C10276) and then washed with PBS. Next, the array was washed with PBS and the modified AAV2-N587UAA_mCherry/AAV-DJ-N589UAA_mCherry was added to each well, incubated at room temperature for 30 minutes and then washed with PBS. Finally, HEK293T cells were added to each well. Cells were imaged for mCherry expression 48 hours post transduction.

#### Lipofectamine coat and neutralization

Oligo A was linked to AAV-DJ-N589UAA_mCherry/ AAV-DJ-N589UAA_gT2 NCas9/ AAV-DJ-N589UAA_CCas9 using the method mentioned above. The modified virus was then incubated at room temperature for 15 minutes with lipofectamine 2000 (ThermoFisher Scientific) in OptiMEM. This was then subjected to neutralization using different dilutions of Pig Serum (Sigma: P9783) in a 50 μl volume made up with DMEM and incubated at 37 °C for one hour. The entire volume after neutralization was added to one well of a 48 well plate with HEK293Ts. The cells were imaged for mCherry expression and flow cytometry analysis was performed 48 hours post transduction and 20,000 cells were analyzed using a FACScan Flow Cytometer and the Cell Quest software (both Becton Dickinson). For the editing experiment, 1E + 5 vg/cell of each AAV-DJ-N58UAA9_gT2 NCas9 and AAV-DJ-N589UAA_CCas9 was used. Cells were harvested 72 hours post transduction for analysis. For the experiment involving Triton X-100, the AAVs were incubated with 0.075% Triton X-100 for 10 minutes before addition onto HEK293T cells. The CCK8 viability assay was carried out 24 hours post transduction of HEK293T cells with AAV-DJ-N589UAA and AAV-DJ-N589UAA + oligo + lipofectamine.

#### Electrophoric Mobility Shift Assay (EMSA) and Western blot

Oligo A was linked to 2E + 11 vg of AAV-DJ-N589UAA (AAV-DJ served as the negative control) via the process of click chemistry (Click-iT – ThermoFisher Scientific, C10276) and then washed with PBS. For EMSA, the samples were denatured and run on a 4–15% gel and stained with coomassie blue. For the western blot, the non-denatured samples were run on a 4–15% gel and transferred to a nitrocellulose membrane. The membrane was first probed with an OligoA’-Biotin conjugate, incubating it at room temperature for 1 hour, followed by 3 TBST washes and the addition of a streptavidin-HRP, incubating it for one hour.

### Statistical Analysis

All statistical analyses were performed using the software Graphpad Prism and p-values were computed by unpaired two-tailed t tests.

### Data availability statement

Any data associated with the study (if not already available in the manuscript and supplementary information) is available from the authors upon request.

## Electronic supplementary material

Supplementary Information
