# Supplementary material for: Oligonucleotide conjugated multi-functional adeno-associated viruses
Source: Sci Rep. 2018 Feb 26;8:3589. doi: 10.1038/s41598-018-21742-x (PMC5827683; doi:10.1038/s41598-018-21742-x)
Supplement: Supplementary file 1 — Supplementary Information [file 41598_2018_21742_MOESM1_ESM.pdf]

SUPPLEMENTARY INFORMATION

Oligonucleotide conjugated multi-functional adeno-associated viruses

Dhruva Katrekar, Ana M. Moreno, Genghao Chen, Atharv Worlikar, Prashant Mali[†]

Department of Bioengineering, University of California San Diego, CA, USA.

[†]*Correspondence:* pmali@ucsd.edu

Section	Page
**SI Figure 1.** UAA structure and incorporation.	2
**SI Figure 2.** Optimization of UAA incorporation into AAVs.	3
**SI Figure 3.** Engineering 'cloaked AAVs'.	4
**SI Figure 4.** Transduction efficiency and cytotoxicity of 'cloaked AAVs'.	5
**SI Figure 5.** Cas9 incorporation into UAA engineered AAVs.	6
**SI Table 1.** List of oligonucleotide sequences.	7
**SI Notes.** Modules for UAA incorporation into AAV2 and AAV-DJ.	8-9

a

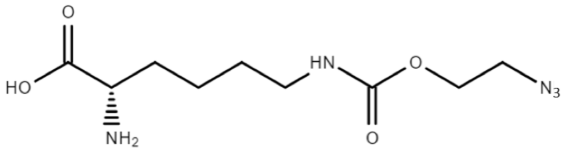

N-epsilon-((2-azidoethoxy) carbonyl)-L-lysine

b

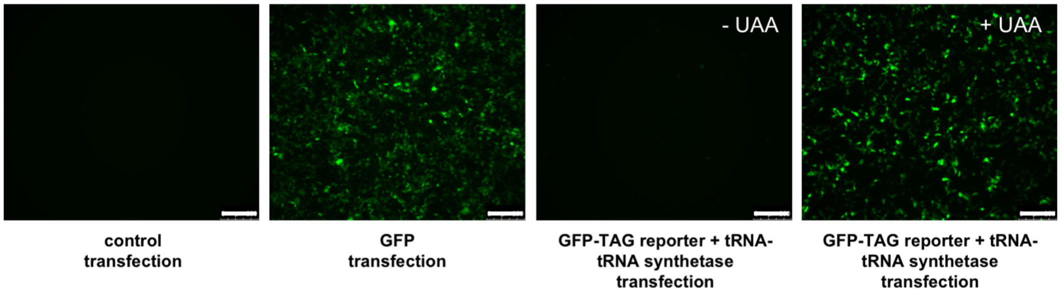

Supplementary Figure 1. UAA structure and incorporation: (a) The structure of N-epsilon-((2-Azidoethoxy)carbonyl)-L-lysine (UAA). **(b)** UAA incorporation into a GFP reporter sequence bearing a TAG stop site at Y39: Fluorescence images of HEK 293T cells 48 hours post transfection are depicted under different experimental conditions: negative control, wt-GFP transfection, and GFP-Y39TAG reporter cum tRNA and tRNA synthetase transfection in the absence or presence of 2mM UAA. UAA incorporation in the latter condition restores robust GFP expression.

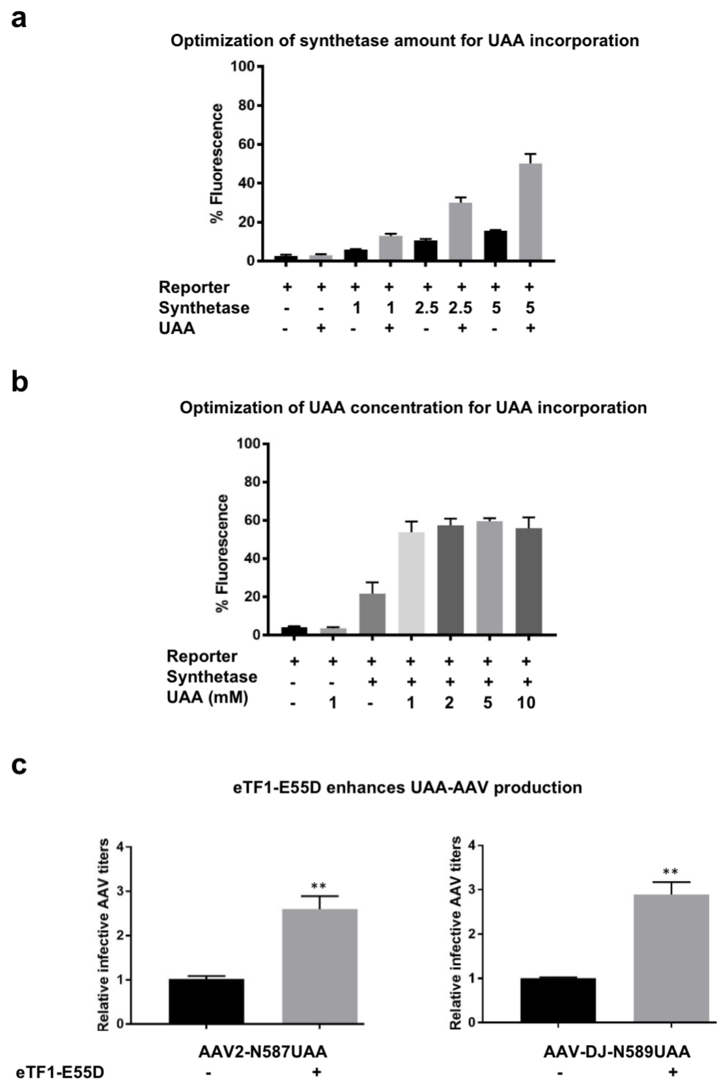

Supplementary Figure 2. Optimization of UAA incorporation into AAVs: (a) Role of synthetase amount on UAA incorporation: optimization of the amount of tRNA and tRNA synthetase plasmid relative to the reporter plasmid (2mM UAA) was performed. A 5:1 mass ratio showed nearly 5-fold higher UAA incorporation as compared to a 1:1 mass ratio of tRNA and tRNA synthetase to the reporter plasmid (error bars are SEM). **(b)** Optimization of UAA concentration on UAA incorporation: a range of UAA concentrations in the presence of 5:1 mass ratio of tRNA and tRNA synthetase to the reporter plasmid were evaluated. No significant difference in incorporation efficiencies was observed, although at high concentrations of UAA there was greater cell death observed in the cultures (error bars are SEM). **(c)** In the presence of eTF1-E55D a 3-fold increase in UAA-AAV titers was observed for both AAV2 and AAV-DJ (error bars are SEM).

a

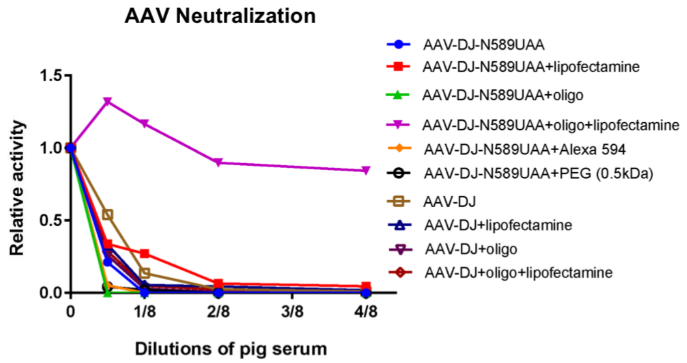

b

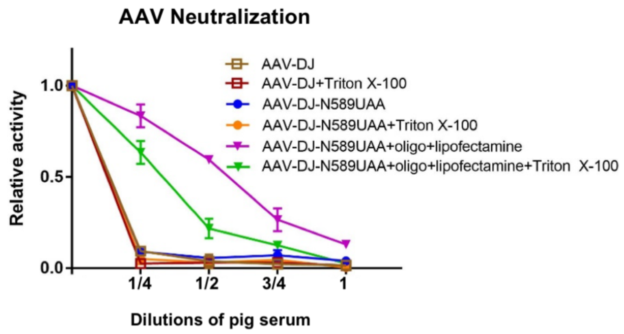

Supplementary Figure 3. Engineering 'cloaked' AAVs: (a) Relative activity of AAV-DJ and AAV-DJ-N589UAA viruses tethered to a range of small molecule and polymer moieties post exposure to pig serum assayed via AAV-mCherry based transduction of HEK 293T. **(b)** Relative activity of AAV-DJ, AAV-DJ-N589UAA and AAV-DJ-N589UAA+oligo+lipofectamine viruses post incubation with 0.075% Triton X-100 followed by pig serum assayed via AAV-mCherry based transduction of HEK 293T cells (error bars are SEM).

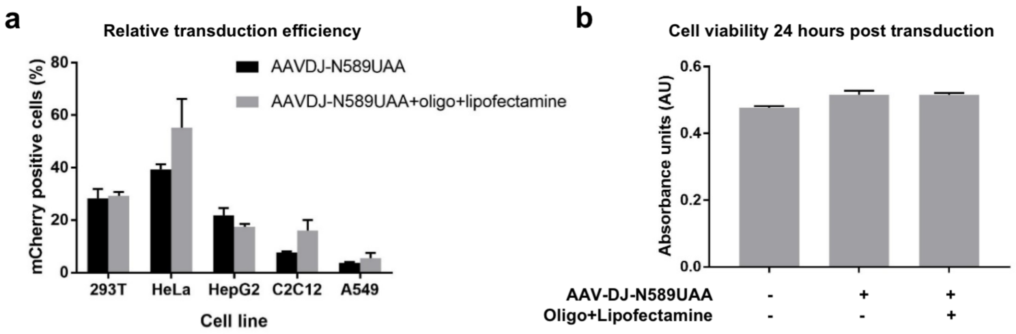

Supplementary Figure 4. Transduction efficiency and cytotoxicity of 'cloaked AAVs': (a) Transduction efficiency of the AAV-DJ-N589UAA and AAV-DJ-N589UAA+oligo+lipofectamine across a variety of cell lines (error bars are SEM). **(b)** Cell viability of HEK 293Ts measured via the CCK8 assay 24 hours post transduction with AAV-DJ-N589UAA and AAV-DJ-N589UAA+oligo+lipofectamine (the absorbance for the CCK8+Media control was 0.17 AU) (error bars are SEM).

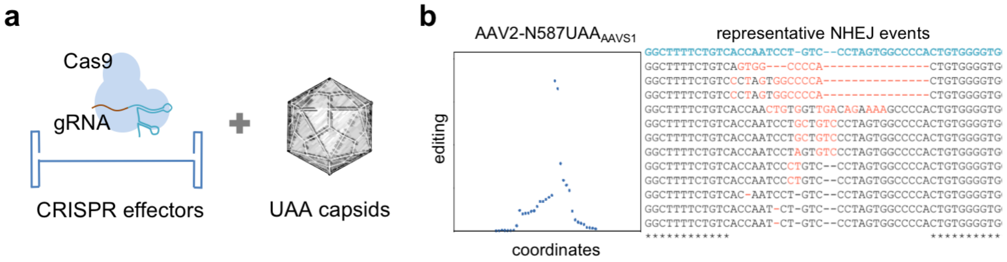

Supplementary Figure 5. Cas9 incorporation into UAA engineered AAVs: (a) Schematic of a Cas9 packaged into a UAA-AAV. **(b)** Confirmation that the UAA modified AAVs can incorporate the split-Cas9 based genome engineering payloads via the ability to effect robust genome editing: indel signature and representative NHEJ profiles are shown (for AAVS1 locus targeting in HEK 293Ts).

Supplementary Table 1. List of oligonucleotide sequences.

a. AAV pseudotyping.

Name	Sequence (5' to 3')	5' group
A	AAAAACTATATTACCCTGTTATCCCTAGCGTAACT	Hexynyl
B	AAAAATATAAGCGGGAGATTCGTCCTCATA	Hexynyl
A'	AGTTACGCTAGGGATAACAGGGTAATATAG	Biosg
B'	TATGAGGACGAATCTCCCGCTTATA	Biosg

b. AAVS1 targeting.

Spacer	GGGGCCACTAGGGACAGGAT
NGS_F	ACACTCTTTCCCTACACGACGCTCTTCCGATCT CGGTTAATGTGGCTCTGGTTCTGG
NGS_R	GACTGGAGTTCAGACGTGTGCTCTTCCGATCT GGGGTTAGACCCAATATCAGGAGACTAG

Supplementary Notes: Modules for UAA incorporation into AAV2 and AAV-DJ.

AAV2 VP1

MAADGYLPDWLEDTLSEGIRQWWKLKPGPPPPKPAERHKDDSRGLVLPGY

KYLGPFNGLDKGEPVNEADAAALEHDKAYDRQLDSGDNPYLKYNHADAEF

QERLKEDTSFGGNLGRAVFQAKKRVLEPLGLVEEPVKTAPGKKRPVEHSP

VEPDSSSGTGKAGQQPARKRLNFGQTGDADSVPDPQPLGQPPAAPSGLGT

NTMATGSGAPMADNNEGADGVGNSSGNWHCDSTWMGDRVITTSTRTWALP

TYNNHLYKQISSQSGASNDNHYFGYSTPWGYFDFNRFHCHFSPRDWQRLI

NNNWGFRPKRLNFKLFNIQVKEVTQNDGTTTIANNLTSTVQVFTDSEYQL

PYVLGSAHQGCLPPFPADVFMVPQYGYLTLNNGSQAVGRSSFYCLEYFPS

QMLRTGNNFTFSYTFEDVPFHSSYAHSQSLDRLMNPLIDQYLYYLSRTNT

PSGTTTQSRLQFSQAGASDIRDQSRNWLPGPCYRQQRVSKTSADNNNSEY

SWTGATKYHLNGRDSLVNPGPAMASHKDDEEKFFPQSGVLIFGKQGSEKT

NVDIEKVMITDEEEIRTTNPVATEQYGSVSTNLQRGNRQAATADVNTQGV

LPGMVWQDRDVYLQGPIWAKIPHTDGHFHPSPLMGGFGLKHPPPQILIKN

TPVPANPSTTFSAAKFASFITQYSTGQVSVEIEWELQKENSKRWNPEIQY

TSNYNKSVNVDFTVDTNGVYSEPRPIGTRYLTRNL

R447; S578; N587; S662

AAV-DJ VP1

MAADGYLPDWLEDTLSEGIRQWWKLKPGPPPPKPAERHKDDSRGLVLPGY

KYLGPFNGLDKGEPVNEADAAALEHDKAYDRQLDSGDNPYLKYNHADAEF

QERLKEDTSFGGNLGRAVFQAKKRLLEPLGLVEEAAKTAPGKKRPVEHSP

VEPDSSSGTGKAGQQPARKRLNFGQTGDADSVPDPQPIGEPPAAPSGVGS

LTMAAGGGAPMADNNEGADGVGNSSGNWHCDSTWMGDRVITTSTRTWALP

TYNNHLYKQISNSTSGGSSNDNAYFGYSTPWGYFDFNRFHCHFSPRDWQR

LINNNWGFRPKRLSFKLFNIQVKEVTQNEGTKTIANNLTSTIQVFTDSEY

QLPYVLGSAHQGCLPPFPADVFMIPQYGYLTLNNGSQAVGRSSFYCLEYF

PSQMLRTGNNFQFTYTFEDVPFHSSYAHSQSLDRLMNPLIDQYLYYLSRT

QTTGGTTNTQTLGFSQGGPNTMANQAKNWLPGPCYRQQRVSKTSADNNNS

EYSWTGATKYHLNGRDSLVNPGPAMASHKDDEEKFFPQSGVLIFGKQGSE

KTNVDIEKVMITDEEEIRTTNPVATEQYGSVSTNLQRGNRQAATADVNTQ

GVLPGMVWQDRDVYLQGPIWAKIPHTDGHFHPSPLMGGFGLKHPPPQILI

KNTPVPADPPTTFNQSKLNSFITQYSTGQVSVEIEWELQKENSKRWNPEI

QYTSNYYKSTSVDFAVNTEGVYSEPRPIGTRYLTRNL

N589

eTF1-E55D

MADDPSAADRNVEIWKIKKLIKSLEAARGNGTSMISLIIPPKDQISRVAK

MLADDFGTASNIKSRVNRLSVLGAITSVQQRLKLYNKVPPNGLVVYCGTI

VTEEGKEKKVNIDFEPFKPINTSLYLCDNKFHTEALTALLSDDSKFGFIV

IDGSGALFGTLQGNTREVLHKFTVDLPKKHGRGGQSALRFARLRMEKRHN

YVRKVAETAVQLFISGDKVNVAGLVLAGSADFKTELSQSDMFDQRLQSKV

LKLVDISYGGENGFNQAIELSTEVLSNVKFIQEKKLIGRYFDEISQDTGK

YCFGVEDTLKALEMGAVEILIVYENLDIMRYVLHCQGTEEEKILYLTPEQ

EKDKSHFTDKETGQEHELIESMPLLEWFANNYKKFGATLEIVTDKSQEGS

QFVKGFGGIGGILRYRVDFQGMEYQGGDDEFFDLDDY
